# Age-adjusted impact of prior COVID-19 on SARS-CoV-2 mRNA vaccine response

**DOI:** 10.3389/fimmu.2023.1087473

**Published:** 2023-01-19

**Authors:** Sachie Nakagama, Yu Nakagama, Yuko Komase, Masaharu Kudo, Takumi Imai, Evariste Tshibangu-Kabamba, Yuko Nitahara, Natsuko Kaku, Yasutoshi Kido

**Affiliations:** ^1^ Department of Virology & Parasitology, Graduate School of Medicine, Osaka Metropolitan University, Osaka, Japan; ^2^ Research Center for Infectious Disease Sciences, Graduate School of Medicine, Osaka Metropolitan University, Osaka, Japan; ^3^ Department of Respiratory Internal Medicine, St. Marianna University, Yokohama Seibu Hospital, Yokohama, Japan; ^4^ Department of Medical Statistics, Graduate School of Medicine, Osaka Metropolitan University, Osaka, Japan

**Keywords:** SARS-CoV-2 infection, vaccination, humoral immunity, antibody, hybrid immunity

## Abstract

More people with a history of prior infection are receiving SARS-CoV-2 vaccines. Understanding the level of protection granted by ‘hybrid immunity’, the combined response of infection- and vaccine-induced immunity, may impact vaccination strategies through tailored dosing. A total of 36 infected (‘prior infection’) and 33 SARS-CoV-2 ‘naïve’ individuals participated. Participants provided sera six months after completing a round of BNT162b2 vaccination, to be processed for anti-spike antibody measurements and the receptor binding domain-ACE2 binding inhibition assays. The relationships between antibody titer, groups and age were explored. Anti-spike antibody titers at 6 months post-vaccination were significantly higher, reaching 13- to 17-fold, in the ‘prior infection’ group. Semi-log regression models showed that participants with ‘prior infection’ demonstrated higher antibody titer compared with the ‘naïve’ even after adjusting for age. The enhancement in antibody titer attributable to positive infection history increased from 8.9- to 9.4- fold at age 30 to 19- to 32-fold at age 60. Sera from the ‘prior infection’ group showed higher inhibition capacity against all six analyzed strains, including the Omicron variant. Prior COVID-19 led to establishing enhanced humoral immunity at 6 months after vaccination. Antibody fold-difference attributed to positive COVID-19 history increased with age, possibly because older individuals are prone to symptomatic infection accompanied by potentiated immune responses. While still pending any modifications of dosing recommendations (i.e. reduced doses for individuals with prior infection), our observation adds to the series of real-world data demonstrating the enhanced and more durable immune response evoked by booster vaccinations following prior infection.

## Introduction

As the cumulative incidence of COVID-19 increases worldwide, more people with a history of prior infection are now receiving SARS-CoV-2 vaccines. With the infection-induced and vaccine-induced immune responses having different viral neutralizing characteristics (1), the acquisition of such a combined immune response is drawing attention as ‘hybrid immunity’. Understanding the role of the combined response of infection- and vaccine-induced immunity in the immune protection of an individual against COVID-19 infection, or in the inhibition of SARS-CoV-2 community transmission, may impact future vaccination strategies through tailored dosing.

With immunopotentiation through repeat vaccinations becoming a pivotal strategy, a consensus ought to be reached on the target population, optimal interval, and dosing regimen for the repeated boosters. To accomplish this, it is becoming increasingly important to understand the longitudinal evolution of the antibody response and the resulting ‘residual immunity’ following vaccination dose(s). The impact of prior infection on the acquisition of protective immunity in vaccinated individuals has been actively studied since the introduction of the SARS-CoV-2 vaccines (2). However, possibly due partly to adherence challenges, many studies have focused on the differences in the early-phase post-vaccine response between naïve and previously infected individuals (3, 4), whereas fewer studies have described this in the mid- to long-term.

We previously carried out a SARS-CoV-2 seroprevalence survey targeting healthcare workers (HCWs) from a tertiary care hospital in Japan. This revealed a nosocomial cluster infection accumulating to a 15.5% overall seroprevalence among the personnel (5, 6). Through longitudinal follow-up and further serological description of the cohort of HCWs (7), we took advantage of the opportunity to investigate a uniformly conditioned population endowed with the combined response of infection- and vaccine-induced immunity: those infected through a nosocomial cluster infection, and later administered the BNT162b2 vaccine through the nation’s mass vaccination campaign following similar intervals after the infection. The impact of prior COVID-19 on an individual’s long-term residual antibody titer following vaccination was analyzed.

## Materials and methods

### Participants and serum sampling

The participants in this study were HCWs at the St. Marianna University, Yokohama Seibu Hospital, Kanagawa, Japan, where we previously conducted an anti-SARS-CoV-2 seroprevalence survey in June 2020 (5). In the previous study, 64 COVID-19-affected HCWs and 350 non-infected individuals were identified following an outbreak having occurred in the hospital during April–May 2020. It was reasonably concluded that all participants had been infected through the cluster infection, given that the SARS-CoV-2 seroprevalence in Japan stayed as low as 0.1% until June 2020 and the close monitoring of symptoms and appropriate testing of the HCWs would have identified any potential symptomatic SARS-CoV-2 infection. From the cohort, 36 individuals who had tested positive (‘prior infection’) and 33 individuals who had tested negative (‘naïve’) on Roche Elecsys anti-SARS-CoV-2 (Roche Diagnostics, Rotkreuz, Switzerland) antibody testing agreed to participate in this follow-up study. The ‘naïve’ individuals were further confirmed to have negative anti-nucleocapsid serology upon study entry. Those categorized as the ‘prior infection’ group, as HCWs, were kept under continuous health monitoring and were confirmed to have had no signs or symptoms indicative of COVID-19 re-infection since completion of the previous survey until their enrollment in this present study.

Participants received two doses of the BNT162b2 vaccine at the standard three-week interval during April–May 2021, and provided their sera six months after completion of their second BNT162b2 dose (two exceptional cases; each were vaccinated in June and July 2021, and thus provided their sera four and five months after completion). The donated sera were processed for anti-spike antibody titer measurements and receptor binding domain (RBD)-ACE2 binding inhibition assays (**Supplementary Figure 1**).

The study was approved by the Osaka Metropolitan University Institutional Ethics Committee [#2020-003]. Written consent for participation was obtained from every participant.

### Assessment of anti-spike and anti-nucleocapsid humoral immunity

The anti-spike antibody titer was measured using two fully automated, commercially available immunoassay platforms. The chemiluminescence immunoassay, Abbott SARS-CoV-2IgG II Quant (Abbott Laboratories, IL, USA), was designed to detect serum IgG antibodies targeting the spike protein of SARS-CoV-2. The electrochemiluminescence (ECL) immunoassay, Roche Elecsys anti-SARS-CoV-2 S (Roche Diagnostics, Rotkreuz, Switzerland), was designed to detect serum total antibodies targeting the spike protein. Sera were also tested for the presence of anti-nucleocapsid antibodies using the ECL immunoassay, Roche Elecsys anti-SARS-CoV-2 (Roche Diagnostics, Rotkreuz, Switzerland). The dual-antigen binding assay detecting total antibodies targeting the nucleocapsid protein was selected for its high sensitivity (8). The assays were performed according to the manufacturers’ instructions.

### Evaluation of the RBD-ACE2 binding inhibition capacity of anti-SARS-CoV-2 antibodies

1:10 diluted serum samples were tested with the Meso Scale Discovery RBD-ACE2 binding inhibition assay, an ECL-labeled competition immunoassay. The V-PLEX SARS-CoV-2 Panel 22 (ACE2) Kit (K15562U) (Meso Scale Diagnostics LLC, MD, USA), containing spots coated with Wuhan, Alpha, Beta, Delta, Gamma, and Omicron RBD antigens, evaluated the capacity of serum anti-SARS-CoV-2 antibodies to inhibit the RBD-ACE2 binding. The ECL signal, negatively proportional to the concentration of inhibitory antibodies in the sample, was read on the MESO QuickPlex SQ 120MM instrument (Meso Scale Diagnostics LLC). RBD-ACE2 binding inhibition capacity was calculated from the following formula and was expressed as ‘Inhibition rate (%Inhibition)’: %Inhibition = {1 – (ECL signal of sample)/(ECL signal of blank)} × 100 [%].

### Statistical analysis

Participants’ demographics were described as numbers (and/or percentages) for categorical variables and as means ± standard deviation for continuous variables, and were compared between ‘naïve’ and ‘prior infection’ groups by the chi-square test or the Mann-Whitney’s U test. The antibody titer was expressed as geometric mean titer (GMT) [95% confidence interval] and compared between groups by the t-test on a logarithmic scale. The relationships between antibody titer, groups (‘naïve’ and ‘prior infection’) and age were explored using semi-log regression models. The age-specific titer ratios were calculated from fitted titer estimates based on t-distribution. The dimorphism of age effect on the log-transformed post-vaccination antibody titer was examined by comparing the interaction terms between groups and age (i.e. the slopes of the semi-log regression lines) with the F-test in ANCOVA. The distributions of %Inhibition in ‘naïve’ and ‘prior infection’ groups were expressed as medians [interquartile ranges] and compared by the Mann-Whitney’s U test. P-values less than 0.05 were considered statistically significant.

## Results

A total of 69 participants (33 categorized as the ‘naïve’ group and 36 as the ‘prior infection’ group) were included in the analysis (**Supplementary Figure 1**). The cohort had a sex ratio of 87% female (88% in ‘naïve’ vs. 86% in ‘prior infection’; P = 0.83) and a mean age of 42 ± 12 years (47 ± 9 years in ‘naïve’ vs. 37 ± 12 years in ‘prior infection’; P = 0.0005) (**Supplementary Table**). Participants self-reported no pre-existing medical conditions known to critically affect antibody response towards any vaccine (i.e. diabetes mellitus, malignant disease, chronic kidney disease). Within the ‘prior infection’ group, the previous COVID-19 diagnosis was often a mild-to-moderate illness, except for a single case of severe disease. Anti-nucleocapsid antibodies remained negative in all ‘naïve’ throughout and remained above the positivity threshold in all of those with ‘prior infection’ except one who had sero-reverted at 6 months post-vaccination.

**Figure 1:**
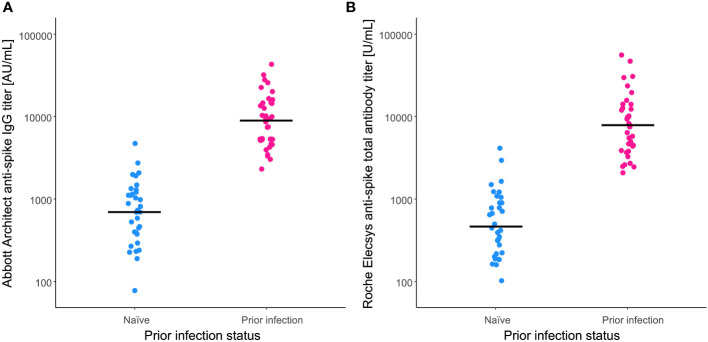
Anti-spike antibody titers after BNT162b2 vaccination. Anti-spike antibody titers were measured at 6 months post-vaccination by the **(A)** Abbott Architect anti-spike IgG assay and the **(B)** Roche Elecsys anti-spike total antibody assay. For comparison, antibody titers of the naïve individuals (blue) and those with a prior infection (pink) are plotted. Solid lines indicate the geometric means of titers.

Compared with the ‘naïve’ group, anti-spike antibody GMT at 6 months post-vaccination were significantly higher in the ‘prior infection’ group (**Figure 1**) (Abbott Architect anti-spike IgG titer 710 [537–939] vs. 9123 [6982–11921] AU/mL; P < 0.0001, Roche Elecsys anti-spike total antibody titer 480 [345–669] vs. 8168 [5945–11222] U/mL; P < 0.0001). For each immunoassay, there was an approximate 13- and 17-fold change, respectively, in the GMT ratio between groups.

**Figure 2:**
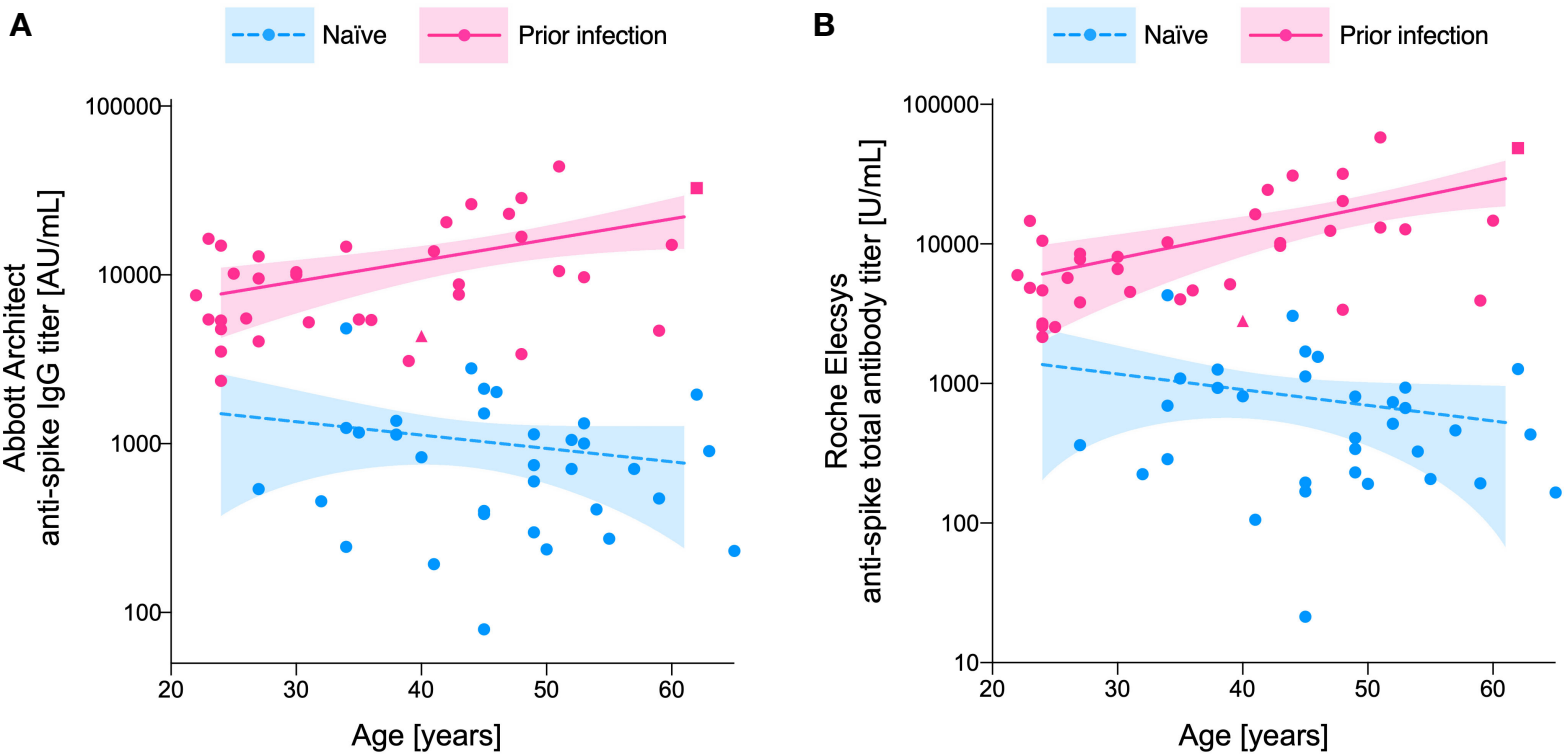
Age-dependent increase in the between-group (‘prior infection’ vs. ‘naïve’) differences in post-vaccination anti-spike antibody titer. Anti-spike antibody titers measured by the **(A)** Abbott Architect anti-spike IgG assay and the **(B)** Roche Elecsys anti-spike total antibody assay are plotted for the naïve individuals (blue) and those with a prior infection (pink). The impact of age on the log-transformed anti-spike antibody titer is fitted with semi-log regression models. Dashed (naïve) and solid (prior infection) lines represent the predicted anti-spike antibody titer calculated by the model, with their 95% confidence intervals represented as shadowed areas. The participants with severe COVID-19 disease (n=1), and sero-reversion of anti-nucleocapsid antibody at the time of serum analysis (n=1), are individually marked in square and triangular symbols, respectively.

Age was positively associated with post-vaccination antibody titer in the ‘prior infection’ group (Spearman’s correlation coefficients: 0.38 (P = 0.022) and 0.52 (P = 0.001) for Abbott and Roche titers, respectively), whereas no such positive correlation was apparent in the ‘naïve’ group (Spearman’s correlation coefficients: -0.20 (P = 0.275) and -0.25 (P = 0.162) for Abbott and Roche titers, respectively). Therefore, the impact of age on the differences in post-vaccination antibody titers was compared between the groups. Evaluated from semi-log regression models (**Figure 2**), the dimorphic effect of age on the log-transformed post-vaccination antibody titer was significant (P = 0.049 and 0.007, for Abbott and Roche titers, respectively; F-test in ANCOVA). Interpolation from the regression models showed that the fold change in the ratio of fitted titer estimates increased from 8.9-fold at age 30 years to 19-fold at age 60 years for the Abbott IgG titer, and 9.4-fold at age 30 years to 32-fold at age 60 years for the Roche total antibody titer (**Table 1**).

**Table 1:** Age-specific differences in anti-spike antibody titers attributable to prior infection status.

Age, y	Titer estimate ratio^a^ (Abbott)	[95% CI^b^]	P-value	Titer estimate ratio^a^ (Roche)	[95% CI^b^]	P-value
Overall	12.8	[8.7–18.9]	< 0.001	17.0	[10.8–26.9]	< 0.001
30	8.9	[4.7–16.8]	< 0.001	9.4	[4.6–19.5]	< 0.001
40	11.5	[6.2–21.3]	< 0.001	14.1	[7.0–28.6]	< 0.001
50	14.8	[7.6–29.2]	< 0.001	21.1	[9.7–45.7]	< 0.001
60	19.1	[8.7–42.3]	< 0.001	31.5	[12.7–78.0]	< 0.001

^a^Ratio (‘prior infection’ to ‘naïve’) of titers fitted from semi-log regression lines.

^b^confidence interval.

**Figure 3:**
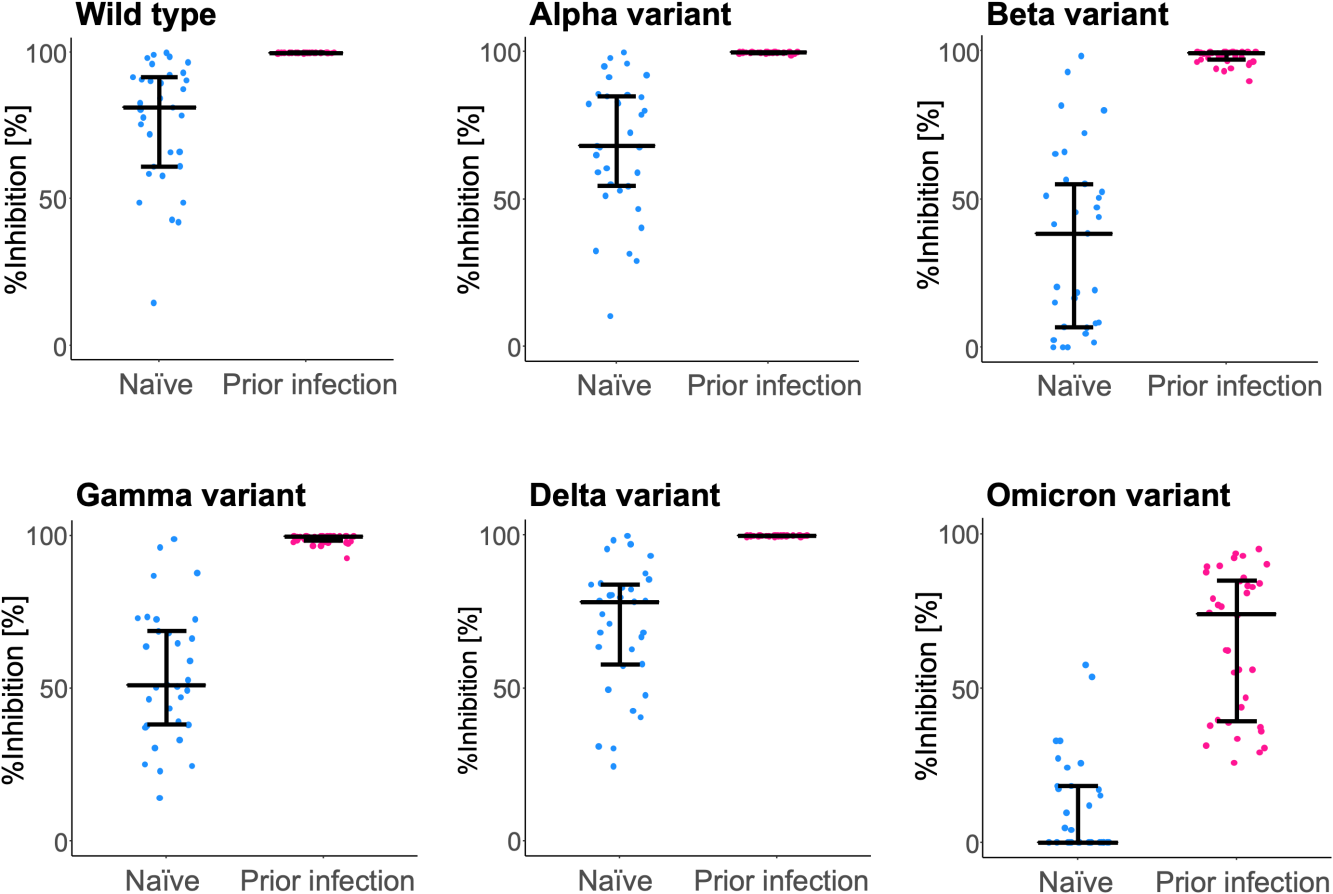
RBD-ACE2 binding inhibition capacity of serum antibodies after BNT162b2 vaccination. Inhibition capacity against the wild type and the variant (Alpha, Beta, Gamma, Delta, and Omicron) SARS-CoV-2 spike antigen was assessed at 6 months post-vaccination. For comparison, inhibition rates of the naïve individuals (blue) and those with a prior infection (pink) are plotted. The bars (error bars) indicate medians (interquartile ranges). %Inhibition, inhibition rate.

In the RBD-ACE2 binding inhibition assay (**Figure 3**), sera of participants from the ‘prior infection’ group showed higher inhibition capacity against all six strains, including the wild type (81.1 [61.1–91.5] vs. 99.8 [99.7–99.9] %; P < 0.0001), and the Alpha (68.1 [54.4–84.8] vs. 99.8 [99.6–99.8] %; P < 0.0001), Beta (38.4 [6.9–55.1] vs. 99.2 [97.0–99.5] %; P < 0.0001), Gamma (51.1 [38.1–68.7] vs. 99.6 [98.4–99.8] %; P < 0.0001), Delta (78.2 [57.8–83.9] vs. 99.8 [99.7–99.9] %; P < 0.0001), and Omicron variants (0.0 [0.0–18.3] vs. 74.1 [39.4–84.9] %; P < 0.0001).

## Discussion

The present study showed that prior infection was predictive of enhanced and durable residual immunity against SARS-CoV-2 at 6 months after vaccination. Participants with ‘prior infection’ demonstrated higher antibody titer compared with the ‘naïve’ individual, even after adjusting for age. Interestingly, the magnitude of the difference in the antibody titer between the two groups seemingly increased with older age. The superiority of ‘prior infection’ maximized at age 60 years, showing 19- and 32-fold higher Abbott and Roche antibody titers, respectively.

The IgG response following SARS-CoV-2 BNT162b2 vaccination (i) peaks rapidly within the first 2 months from the initial dose and then (ii) enters a subsequent stage of gradual decay (9). The initial studies reporting the effect of prior infection on BNT162b2 post-vaccination antibody titers had often targeted the peak response. At 2 months and 3 months after the initial dose, 3.7-fold and 2.7-fold increases, respectively, were observed in vaccinees with prior COVID-19 infection compared with the naïve group (3, 4). While potentiation of the peak response to BNT162b2 vaccination by ‘prior infection’ has been well supported by abundant real-world data, the stage of IgG decay has been less addressed. Recently, a modeling study of post-vaccination ‘waning immunity’ showed that the anti-SARS-CoV-2 IgG levels of vaccinees with prior infection decreased at a slower rate compared to the non-previously infected (10). Another study also suggested a slower decay of antibody titers in the prior infection group, resulting in a further exaggerated fold change in titer during the decay phase of antibodies (11). The here observed unexpectedly large 13- to 17-fold change in antibody titers, attributed to prior infection status, is thus fully interpretable considering the biphasic kinetics of the post-vaccination immune evolution.

Interestingly, age had dimorphic effects on post-vaccination immune evolution depending on prior infection status. Older age was associated with a higher level of IgG in previously infected individuals, whereas no such positive correlation was apparent in the naïve group. Older age has been repeatedly observed as a risk factor of attenuated post-vaccination antibody titer in the previously naïve population (9). To the contrary, increasing age has been associated with stronger antibody responses in convalescent plasma donors (12, 13). This can be explained by the fact that older individuals are more prone to symptomatic, and possibly more severe, SARS-CoV-2 infection, which in turn is often accompanied by a potentiated circulating IgG response (5). Our study, analyzing the combined effect of infection- and vaccine-induced antibody responses in the ‘prior infection’ group, has also demonstrated an accentuation of difference in the antibody titer with increasing age. Altogether, it seems that the age effect on prior infection is reflected more strongly in the combined immune status of ‘infection- plus vaccine-induced immunity’. To support this idea, the present cohort of vaccinees with prior infection showed a strong positive correlation between the peak anti-spike antibody response following their COVID-19 diagnosis (at 2 months’ convalescence) and the residual antibody titer at 6 months post-vaccination (Pearson’s correlation coefficient: 0.71 (P < 0.0001) and 0.77 (P < 0.0001) for Abbott and Roche titers, respectively).

Immunopotentiation through repeated boosters is an affordable strategy only when the risk-benefit balance is optimized and deemed favorable. For the influenza vaccine, prior-year vaccination has shown to have negative effects on the current year’s vaccine effectiveness (14). Further, a frequent vaccination history was associated with 41% and 27% decreases in vaccine effectiveness against type A influenza and type B influenza, respectively (15). This phenomenon has been explained as ‘antibody feedback’ (also known as the ‘original antigenic sin’ or ‘immune imprinting’) (16). Potential ‘antibody feedback’ has also been suggested with the SARS-CoV-2 vaccines (17). An extended 3-month interval regimen has resulted in, on average, 3.5-fold higher IgG titers (18). A longer interval between prior infection and boosting of the immune response with a vaccine has been associated with more enhanced and durable immune responses (19). As shown in the present study, the evolution of post-vaccine immune responses differ among those having experienced prior infection compared with the naïve. Thus, non-stratified strategies for repeated boosters may lead to unexpected harms or attenuated performance through the ‘antibody feedback’ mechanism. Further, although limited in evidence and awaiting additional validation data, a study has suggested only a modest dose-dependent (one or two doses) increment on COVID-19 risk reduction for booster vaccinations following prior infection (20). From the risk-benefit balance perspective, when and whom to target with the repeated booster vaccinations remains a crucial question to future vaccination campaigns.

The limitation of the study is the limited number of individuals evaluated. The observed immune response may not represent that of the overall population. The immune response of individuals from older age categories and at utmost risk of severe disease would have been highly intriguing, although not covered in the present study. The extreme elderly and multi-morbid population have been shown to exhibit aberrant immune responses (21, 22).

Despite these limitations, this study provides relevant information in a context where hybrid immunity is becoming increasingly prevalent. The benefits of boosting the infection-acquired immunity by vaccination has been shown ‘clinically’ to enhance the degree and duration of protection (protection rate persistently above 90% for 18 months or longer) (19). The present study, in turn with robust indices of protective antibody response, further enriches the evidence for and provides an immunological basis to this highest and most durable protection achieved by those vaccinated on top of a primary infection. While still pending any modifications of dosing recommendations (i.e. reduced doses for individuals with prior infection), our observation adds to the series of real-world data demonstrating the enhanced and more durable immune response evoked by booster vaccinations following prior infection.

## Data availability statement

The original contributions presented in the study are included in the article/**Supplementary Material**. Further inquiries can be directed to the corresponding author.

## Ethics statement

The studies involving human participants were reviewed and approved by Osaka Metropolitan University Institutional Ethics Committee. The patients/participants provided their written informed consent to participate in this study.

## Author contributions

Conceptualization: SN and YNa. Methodology: SN, YNa, and YKi. Investigation: SN and YKo. Data curation: YNa, YNi, and NK. Formal analysis: MK, TI, and ET-K. Writing-original draft preparation: SN and MK. Writing-review and editing: YNa, TI, ET-K, and YK. Funding acquisition: YNa, NK, and YKi. All authors contributed to the article and approved the submitted version.

## Supplementary material

Supplementary Figure 1 Enrollment, vaccination, and serum sampling of participants. Sixty-nine participants (33 naïve and 36 with prior infection) were included in the analysis and followed the indicated protocol. As indicated in the Materials and methods, vaccination for two cases exceptionally began in June and July 2021, resulting in four and five-month intervals for serum donation, respectively).
