# Supplementary figures and images for: Age-adjusted impact of prior COVID-19 on SARS-CoV-2 mRNA vaccine response
Source: Front Immunol. 2023 Jan 19;14:1087473. doi: 10.3389/fimmu.2023.1087473 (PMC9892832; doi:10.3389/fimmu.2023.1087473)

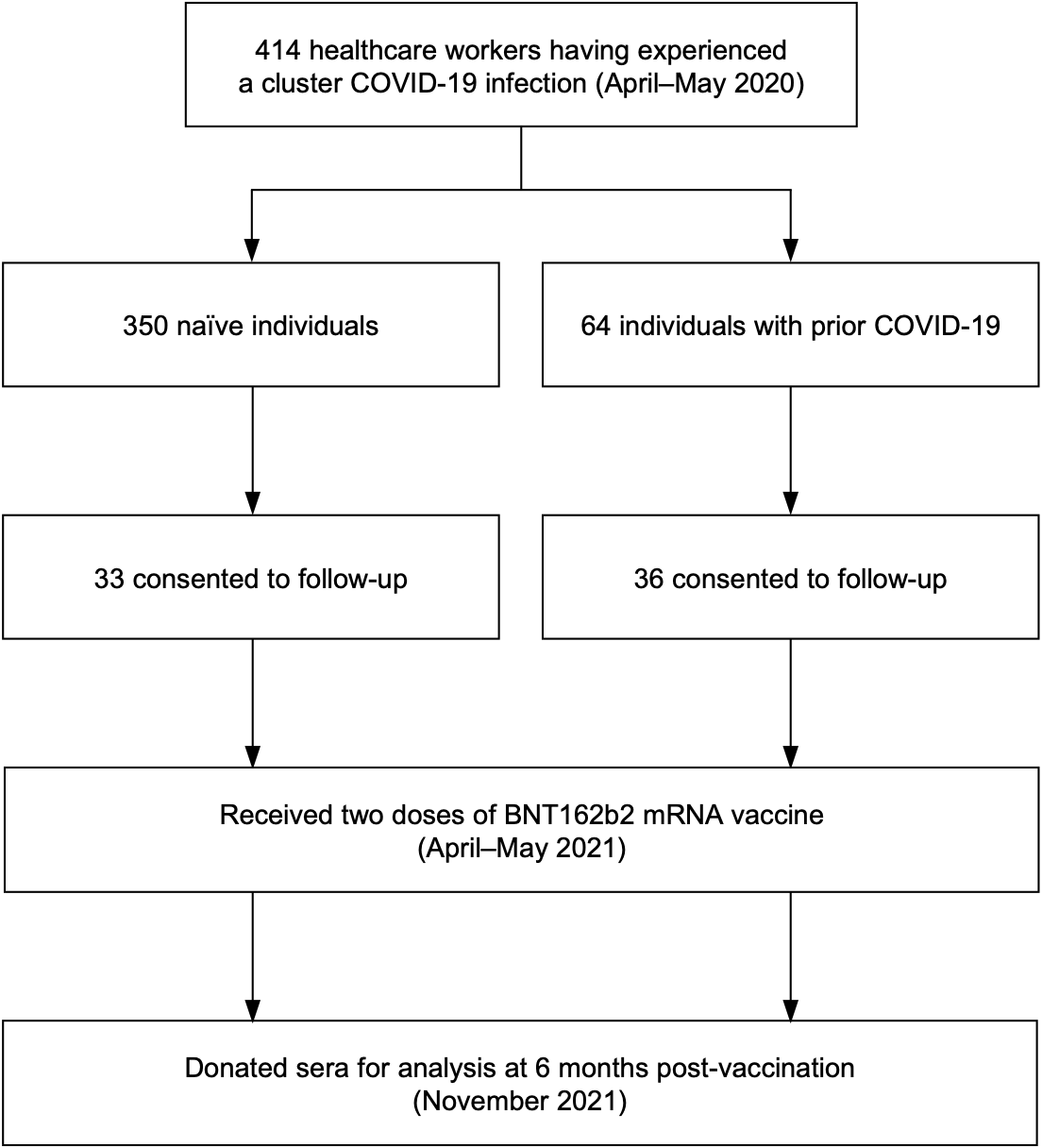

Supplement: Supplementary Figure 1 — Enrollment, vaccination, and serum sampling of participants. Sixty-nine participants (33 naïve and 36 with prior infection) were included in the analysis and followed the indicated protocol. As indicated in the Materials and methods, vaccination for two cases exceptionally began in June and July 2021, resulting in four and five-month intervals for serum donation, respectively). [file Image_1.tiff]
