# Supplementary material for: Age-adjusted impact of prior COVID-19 on SARS-CoV-2 mRNA vaccine response
Source: Front Immunol. 2023 Jan 19;14:1087473. doi: 10.3389/fimmu.2023.1087473 (PMC9892832; doi:10.3389/fimmu.2023.1087473)
Supplement: Supplementary file 2 [file Table_1.docx]

Supplementary Table. Descriptive characteristics of participants

| Variable | Naïve | (n = 33) | Prior infection | (n = 36) | P-value |
| --- | --- | --- | --- | --- | --- |
| Age, y [SD]^a^ | 47 | [9] | 37 | [12] | 0.0005 |
| Sex, female [%] | 29 | [88] | 31 | [86] | 0.83 |
| COVID-19 severity, n [%] |  |  |  |  |  |
| Mild | - | [-] | 30 | [83] | - |
| Moderate | - | [-] | 5 | [14] | - |
| Severe | - | [-] | 1 | [3] | - |

a standard deviation
